# The Development of Secretory Cavities in *Zanthoxylum nitidum* Leaves and the Pattern of Essential Oil Accumulation

**DOI:** 10.3390/plants14223449

**Published:** 2025-11-11

**Authors:** Yang Yang, Jiating Hou, Jiaxin Zeng, Yue Fang, Tao Tian, Xin Wang, Rui Kai, Sisheng Zhang, Weiyao Liao, Tao Chang, Ran Zheng, Yang Chen, Yanqun Li, Mei Bai, Hong Wu

**Affiliations:** 1 School of Pharmaceutical Sciences, Hunan University of Medicine, Huaihua 418000, China; hnum_yy@163.com (Y.Y.);; 2 Guangdong Laboratory for Lingnan Modern Agriculture, College of Life Sciences, South China Agricultural University, Guangzhou 510642, China; 3 Biomedical Research Institute, Hunan University of Medicine, Huaihua 418000, China

**Keywords:** *Zanthoxylum nitidum*, leaf, essential oil (EO), secretory cavity, gas chromatography-mass spectrometry (GC-MS), fourier transform infrared spectroscopy (FTIR)

## Abstract

The root of *Zanthoxylum nitidum* is used in traditional Chinese medicine, whereas its leaves remain an under-exploited resource rich in essential oil (EO). By integrating cytological, analytical–chemical, and chemometric approaches, we have dissected the ontogeny of secretory cavities and the temporal accumulation of EO in *Z. nitidum* leaves for the first time. Cytological analyses revealed marginal-tooth-slit secretory cavities consisting solely of a spherical domain formed via a schizogenous mechanism. The EO yield followed a unimodal trajectory, peaking at growth stages ZN-2 and ZN-3. Gas chromatography–mass spectrometry (GC-MS) profiling identified 60 constituents; sesquiterpenoids reached maximal abundance at ZN-3, whereas monoterpenoids predominated at ZN-2. Second-derivative Fourier transform infrared spectroscopy (FTIR) spectra exhibited pronounced stage-specific differences, and hierarchical cluster analysis coupled with principal component analysis reliably discriminated developmental stages based on their chemical fingerprints. These findings provide a robust cytological and analytical framework for quality control and rational utilization of *Z. nitidum* leaves, laying the groundwork for their full medicinal exploitation.

## 1. Introduction

*Zanthoxylum nitidum* (Roxb.) DC. is a perennial woody vine in the Rutaceae family, widely used as a traditional Chinese folk medicine for anti-inflammatory and analgesic purposes. In traditional Chinese medicine (TCM), *Z. nitidum* is valued for its effects of promoting blood circulation to remove blood stasis, regulating qi to alleviate pain, dispelling wind to unblock the collaterals, and detoxifying to reduce swelling [1,2]. Its principal bioactive constituents are alkaloids such as nitidine chloride and chelerythrine [2,3,4]. Contemporary clinical and pharmacological studies have demonstrated its anti-inflammatory, analgesic, antimicrobial, antitumor, and cardiovascular activities [2,3,5,6]. *Z. nitidum* is the key raw material for more than 60 Chinese patent medicines, as well as for everyday products such as toothpaste [7,8]. It is therefore one of China’s most economically important natural plant resources.

The Pharmacopeia of the People’s Republic of China (PPRC) stipulates that the medicinal part of *Z. nitidum* is its root [1]. However, classical and modern references such as the Shennong’s Classic of Materia Medica, Mingyi Bielu, Chinese Materia Medica Dictionary, and Modern Compendium of Materia Medica record that its root, stem, leaf, and fruit are all used medicinally [9,10]. As a perennial species, harvesting *Z. nitidum* for its roots alone leads to massive wasteage of the aerial biomass of this medicinal resource, especially the leaves. Studies have shown that the alkaloid levels in the aerial parts are markedly lower than in the roots [11,12], whereas the essential oil (EO) content in the leaves is significantly higher than in both the roots and stems [10]. Vilaysack et al. [13] found that the principal constituents of *Z. nitidum* leaves’ EO remained qualitatively similar across different months. Moreover, numerous studies have confirmed that the EOs of *Z. schinifolium* Siebold and Zucc., *Z. myriacanthum* var. *pubescens* (C. C. Huang) C. C. Huang, and *Z. coreanum* Nakai all exhibit significant anti-inflammatory effects in animal experiments [14,15,16,17]. Our previous work demonstrated that *Z. nitidum* leaf EO possesses markedly higher in vitro antioxidant activity than the root, stem, or pericarp EOs [10]. In addition, the leaf EO inhibits *Bacillus subtilis* (Ehrenberg) Cohn and *Escherichia coli* T. Escherich and exhibits cytotoxic activity against hepatoma, cervical, and human breast carcinoma cells [18]. These findings indicate that *Z. nitidum* leaves are a valuable medicinal resource; their utilization not only prevents wastage of TCM biomass but also enhances the economic returns of cultivation enterprises.

The biosynthesis and accumulation of EOs in plants are intimately linked to the structural characteristics of their specialized secretory organs [19]. For example, in the oil cells of *Cinnamomum cassia* (L.) D. Don [20,21] and *Persea americana* Mill. [22], the secretory cavities in *Citrus grandis* ‘*Tomentosa*’ [23] and *Citrus medica* ‘*Fingered*’ [24], as well as the secretory ducts in *Bupleurum chinense* Franch. [25], all exhibit close developmental relationships with EO secretion.

Secretory cavities are a structural hallmark common to Rutaceae, occurring widely in the stems, leaves, flowers, and fruits. Three modes of cavity ontogeny have been proposed for rutaceous secretory structures: lysigenous, schizogenous, and schizolysigenous [19,26]. Studies have shown that secretory cavities in *Poncirus* and *Citrus* species arise lysigenously [27,28]. In *Tetradium ruticarpum* (A. Juss.) T. G. Hartley, leaf secretory cavities originate from the spongy mesophyll beneath the lower epidermis without the involvement of epidermal cells, and their lumina form schizogenously [29]. In contrast, secretory cavities in *C. sinensis* (L.) Osbeck leaves develop through a schizolysigenous process [30]. Liu and Hu [26] observed that secretory cavities in *Z. nitidum* occur exclusively in the recesses between marginal leaf-teeth and are characterized by large diameters and numerous epithelial cells. Liu et al. [23] demonstrated that in *C. grandis* ‘*Tomentosa*’ fruit, the secretory cavity lumen forms schizolysigenously; these cavities serve as principal sites for the distribution and accumulation of bioactive constituents, with EO content rising progressively as the cavities develop. The mode of initiation and the structural characteristics of secretory cavity formation in *Z. nitidum* leaves, together with the dynamics of EO content and composition, have yet to be reported.

In this study, leaves of *Z. nitidum* at different developmental stages were investigated to elucidate the developmental characteristics and formation mechanism of leaflet secretory cavities using semi-thin sectioning. The EO yield was quantified, and the chemical composition was analyzed using gas chromatography–mass spectrometry (GC-MS) and Fourier transform infrared spectroscopy (FTIR) across developmental stages to clarify the dynamics of secretory cavity morphogenesis and EO accumulation. The findings provide fundamental cytological and analytical data for the quality control and rational utilization of *Z. nitidum* leaves as medicinal materials, offering a scientific basis for their comprehensive development and utilization with the ultimate goal of improving yield per unit area, promoting industrial development, and supporting rural revitalization.

## 2. Materials and Methods

### 2.1. Experimental Materials

Seedlings of *Z. nitidum* (Rutaceae) were collected in November 2021 from a standardized medicinal cultivation base in Yunfu City, Guangdong Province, China (112°3′ E, 22°54′ N), and identified by Professor Hong Wu of South China Agricultural University. After two years of acclimatization and transplantation, sampling experiments were conducted on leaves at different developmental stages from multiple individual plants.

### 2.2. Semi-Thin Sectioning

Our methods followed Yang et al. [31]. Leaves of *Z. nitidum* at different developmental stages were collected and cut into small pieces (0.5 cm × 0.5 cm), then fixed in 2% glutaraldehyde + 3% paraformaldehyde (prepared in 0.1 M PBS, pH 7.2) under vacuum at 4 °C for 12 h. The samples were rinsed three times with PBS (pH 7.2) and post-fixed in 2% osmium tetroxide for 2 h, followed by three additional PBS washes. Dehydration was performed through a graded ethanol series (30%, 50%, 70%, 85%, 90%, 95%, and 100%, for ~10 min each). Tissues were then infiltrated with 100% ethanol/propylene oxide (1:1) for 15 min, followed by two changes of pure propylene oxide (15 min each). The samples were embedded by sequential infiltration with propylene oxide/Epon812 resin (1:1) for 24 h and pure Epon812 resin twice for 12 h each, then polymerized at 60 °C for 24 h. Semi-thin sections (1~2 µm) were cut using a Leica RM2155 microtome (Leica; Nussloch, Germany), stained with 0.5% toluidine blue, and observed and photographed with a Leica DMLB microscope (Leica; Nussloch, Germany).

### 2.3. Extraction and Quantification of EO

Leaves at four developmental stages were air-dried, ground, and passed through a 60-mesh sieve. For each stage, 30 g of leaf powder was placed in a 1000 mL round-bottom flask, mixed with 300 mL of distilled water, shaken, and soaked for 12 h. Essential oils were extracted using aqueous distillation according to the method described in the PPRC [1]. The extracted oils were weighed, and each group of samples was measured in triplicate to determine the average yield. The essential oils were stored at −20 °C until analysis was performed.

### 2.4. GC-MS Analysis

The GC-MS operating conditions were as follows: column—DB-5 MS (30 m × 0.25 µm × 0.25 mm); injector temperature—300 °C; temperature program—initial oven temperature 60 °C held for 4 min, ramped at 8 °C min^−1^ to 100 °C and held for 8 min, ramped at 8 °C min^−1^ to 150 °C and held for 5 min, ramped at 5 °C min^−1^ to 200 °C and held for 2 min, then ramped at 10 °C min^−1^ to 300 °C; carrier gas—high-purity helium at a constant flow rate of 1.0 mL min^−1^.

EO was dissolved in dichloromethane, filtered through a 0.22 µm organic membrane, and 2 µL was injected at a 20:1 split. The solvent delay was 4 min, and the settings were EI = 70 eV, ion source = 200 °C, and m/z = 35~650 amu. Compounds were identified using NIST library matching of their MS spectra and quantified by relative peak area using Xcalibur (Thermo Fisher; Waltham, MA, USA). GC profile: 4.00~50.00 min.

### 2.5. FTIR Analysis

FTIR spectra were acquired on a Nicolet 5700 spectrometer (Thermo Nicolet Corp.; Madison, WI, USA) equipped with a DTGS detector (Thermo Nicolet Corp.; Madison, WI, USA). Spectra were collected over 4000–400 cm^−1^ at 4 cm^−1^ resolution in a room maintained at 25 °C and 30% relative humidity. Dried samples were ground to 200-mesh powder. Exactly 1.00 mg of sample was thoroughly mixed with 100.00 mg dried KBr and pressed into 5 mm × 1 mm pellets. Each sample was scanned 64 times, repeated in triplicate, and the averaged spectrum was used after subtracting ambient air, H_2_O, and CO_2_ contributions [32]. Baseline correction and smoothing were performed with OMNIC 8.2.

### 2.6. Hierarchical Cluster Analysis (HCA)

Hierarchical clustering is a method that constructs a nested grouping of observations. Any accepted similarity (or distance) metric can be employed; consequently, samples within the same cluster are highly similar, whereas those in different clusters are markedly dissimilar [33]. In this study, hierarchical cluster analysis was performed using Pearson correlation. The relative percentages of the common GC-MS peaks served as the variables, and the distance matrix among samples was derived accordingly. Clustering and dendrogram generation were executed in SPSS 20.0 (IBM, New York, NY, USA).

### 2.7. Principal Component Analysis (PCA)

PCA is a spectral-feature extraction technique that, by maximizing variance, linearly recombines the numerous original variables into a reduced set of new, low-dimensional variables [20]. In this study, PCA was performed via singular value decomposition of the second-derivative FTIR data matrix. The first three principal component scores were used to generate projection plots that visually reveal fingerprint similarity. All PCA computations were carried out in SPSS 20.0.

### 2.8. Data Analysis

All data are presented as the mean ± SEM. Statistical analysis was performed using one-way ANOVA followed by Duncan’s multiple-comparison test (SPSS 20.0). *p* < 0.05 was considered statistically significant.

## 3. Results and Discussion

### 3.1. Distribution of Secretory Cavities

**Figure 1:**
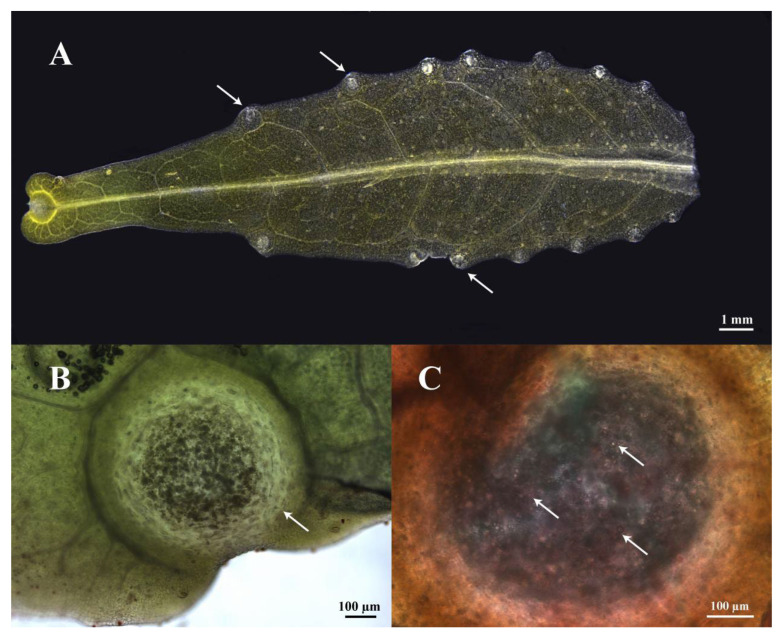
(**A**) Distribution of secretory cavities (white arrows) in *Zanthoxylum nitidum* leaf, bar = 1 mm. (**B**) Secretory cavity, bar = 100 µm. (**C**) Essential oil droplets (white arrows) stained with Sudan red in the secretory cavity, bar = 100 µm.

SEM revealed *Z. nitidum* leaflet secretory cavities to be of the marginal-tooth-slit type: one globular cavity per tooth sinus, large enough to be macroscopically discernible (Figure 1A,B). Sudan red staining showed abundant essential oil droplets within each cavity (Figure 1C). These cavities, universally present in Rutaceae, are the principal sites of volatile oil biosynthesis [26,34,35]. Among the three recognized types, *Z. nitidum*, *Z. planispinum* DC., and *T. glabrifolium* (Champ. ex Benth.) T. G. Hartley all exhibit the marginal-tooth-slit form [26].

### 3.2. Ontogenetic Mode of Secretory Cavities

**Figure 2:**
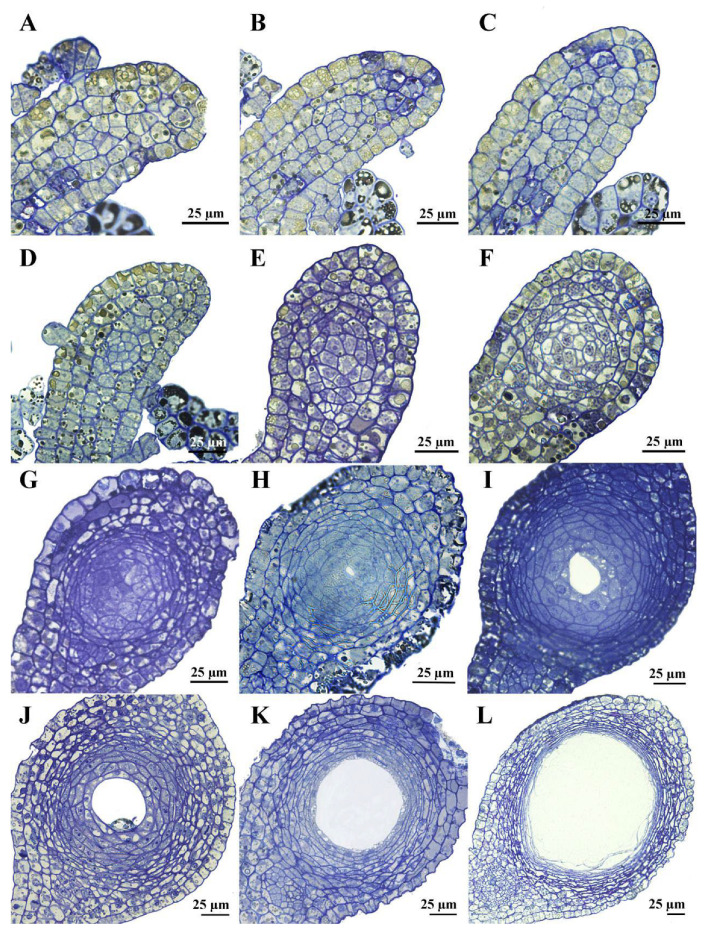
Semi-thin sections of secretory cavity development in *Zanthoxylum nitidum* leaf. (**A**–**F**), initial cell stage; (**G**,**H**), schizogenous stage; (**I**,**J**), cavity expansion stage; (**K**,**L**), maturation stage. Bar = 25 µm.

Secretory cavity development is divided into four phases: initial cell, schizogenous intercellular space formation, cavity expansion, and maturation [23,24,26]. During the initial cell phase, one densely cytoplasmic central cell first undergoes a periclinal division to produce two cells (Figure 2A), then an anticlinal division to yield four (Figure 2B). Subsequent longitudinal or transverse divisions (Figure 2C) generate a 10-to-12-cell circular or elliptical primordium (Figure 2D,E).

During the schizogenous phase, the primordium proliferates and enlarges the cell cluster (Figure 2F). Local wall swelling between 2~3 central cells generates fissures at cell junctions (Figure 2G); these clefts propagate, yielding a micro-cavity encircled by 5~6 cells (Figure 2H). Only the spherical domain was observed in *Z. nitidum*. Central cells remain meristematic with dense cytoplasm, small vacuoles, and large central nuclei [23,24]; two to three isodiametric and cytoplasm-rich inner cell layers become epithelial cells; and a three-to-four-layer outer mantle of smaller, flattened cells differentiates into the sheath [23,24].

Initial cavity expansion is driven by peripheral cells inserting between the inner layers to augment the epithelial cell number. Subsequently, epithelial cells vacuolate and elongate tangentially, rapidly enlarging the lumen (Figure 2I). Continued swelling thins their walls, distorts their shape, and produces a large central vacuole (Figure 2J). Progressive dissolution then proceeds centripetally, leaving only 1~2 epithelial layers intact.

At maturity, the secretory cavity enlarges to a diameter of about 90 µm. A one-to-two-layered sheath of tangentially flattened epithelial cells borders the lumen; these cells exhibit dense cytoplasm and only minute vacuoles. Beyond them, four to six additional layers of even flatter sheath cells display markedly higher vacuolation (Figure 2K,L).

Semi-thin sections revealed that the secretory cavities in *Z. nitidum* leaves consist solely of a globular zone and arise schizogenously. Earlier studies reported that the secretory cavities of *Ruta* are schizogenous, whereas those in *Poncirus* and *Citrus* are lysigenous [27,28,36]. Liu and Hu [37] demonstrated that the secretory cavities in *Z. bungeanum* Maxim. fruit are likewise schizogenous. With improved techniques, a survey of 42 Rutaceous species confirmed that schizogeny is the predominant mode of cavity initiation [26], a finding consistent with our observations in *Z. nitidum*. These cavities serve as the primary storage sites for EOs and are thought to protect leaves against herbivorous insects [38,39].

### 3.3. Comparison of EO Contents

**Figure 3:**
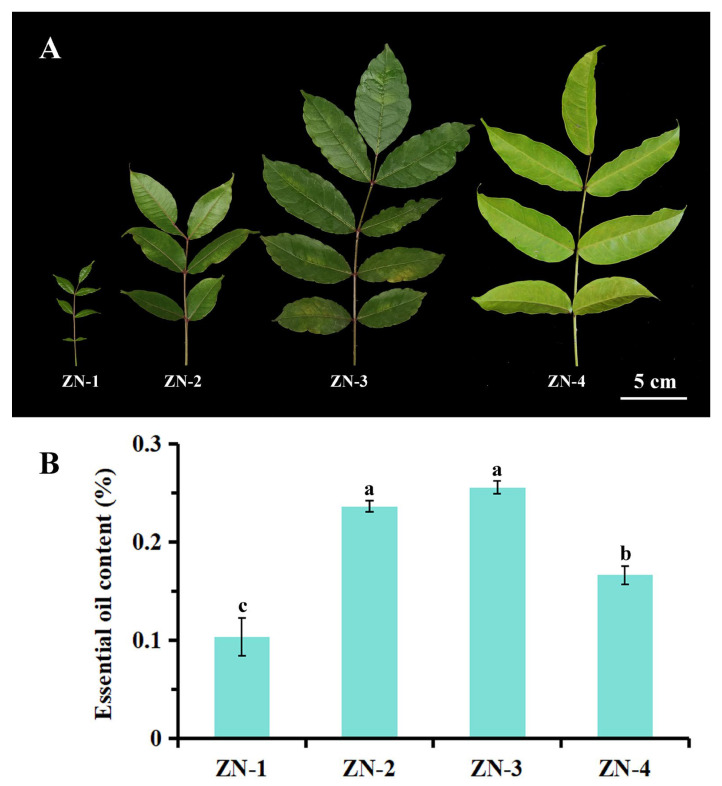
(**A**) *Zanthoxylum nitidum* leaves at different developmental stages, bar = 5 cm. (**B**) Histogram of essential oil yields from leaves at different stages; different lowercase letters “a”, “b”, and “c” indicate significant differences among samples (*p* < 0.05).

EO yields from leaves at different growth stages (Figure 3A) are shown in Figure 3B. The yield initially increased and then declined. Stages ZN-3 and ZN-2 produced the highest yields (0.26% and 0.24%, respectively), with no significant difference between them (*p* > 0.05). The yield at ZN-4 (0.17%) was significantly higher than that at ZN-1 (0.10%). Under the same hydro-distillation method, fresh leaves from Vietnam and Australia yielded only 0.01% and 0.10%, respectively [18,40], whereas those from upper Assam, northeastern India, reached 0.76% [41]. In our previous study, *Z. nitidum* leaves yielded 0.21% [10]. In the present study, yields across stages ranged from 0.10% to 0.26%, peaking at intermediate maturity. During early leaf development, rapid cell division and expansion initiated secondary metabolite accumulation; at full maturity, biosynthesis plateaued. Upon senescence, essential oils and other nutrients were remobilized or degraded, reducing yield [42,43]. Similar trends were reported for *C. cassia* leaf EO, the alkaloids of *Z. nitidum*, and the chlorogenic acid and luteoloside of *Lonicera japonica* Thunb. [2,21,44]. Thus, stages ZN-2 and ZN-3 were identified as the optimal harvest window for essential oil production.

### 3.4. Chemical Composition of the EO

**Figure 4:**
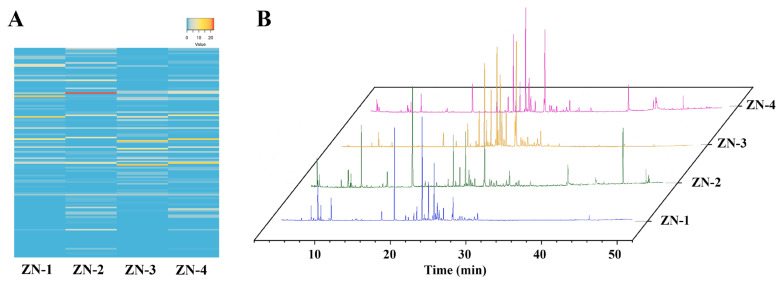
(**A**) Heat map and (**B**) GC-MS chromatograms of essential oils from *Zanthoxylum nitidum* leaves at different developmental stages.

GC-MS analysis of EOs from *Z. nitidum* leaves at four developmental stages (Figure 4A,B) identified a total of 60 constituents (Figure 4B, Table 1) accounting for 88.96~90.36% of the total EO. The number of compounds detected at each stage was 34, 35, 32, and 33 for ZN-1, ZN-2, ZN-3, and ZN-4, respectively. In ZN-1, 2-undecanone (18.00%), β-caryophyllene (15.37%), β-copaene (9.93%), α-humulene (6.01%), and *trans*-nerolidol (4.84%) dominated, giving 54.10% sesquiterpenoids, 18.43% ketones, and 16.18% monoterpenoids. ZN-2 was richest in piperitone (22.47%), followed by *trans*-nerolidol (8.55%), germacrene D (8.35%), linalool (8.17%), and β-caryophyllene (6.51%), with 39.75% monoterpenoids and 37.47% sesquiterpenoids. ZN-3 was characterized by *trans*-nerolidol (17.39%), β-copaene (15.92%), bicyclogermacrene (9.61%), β-caryophyllene (8.78%), and α-humulene (5.61%), resulting in 84.89% sesquiterpenoids and only 2.44% monoterpenoids. In ZN-4, β-copaene (15.87%), *trans*-nerolidol (15.55%), β-caryophyllene (9.80%), bicyclogermacrene (5.87%), and n-hexadecanoic acid (4.81%) were foremost, yielding 66.37% sesquiterpenoids and 9.92% monoterpenoids.

All four stages shared the dominant constituents *trans*-nerolidol, α-humulene, β-caryophyllene, α-cadinol, δ-amorphene, α-farnesene, bicyclogermacrene, linalool, piperitone, and germacrene B, and were dominated by sesquiterpenoids, consistent with our previous report of 68.50% sesquiterpenoids in *Z. nitidum* leaf oil [10]. In contrast, *Z. nitidum* leaf EO in Australia is predominantly composed of sesquiterpenes (35.80%), whereas the leaves from Vietnam and India mainly contain monoterpenoids, at 62.10% and 60.00%, respectively [18,40,41]. The type and abundance of secondary metabolites in a medicinal plant vary with growth environment, developmental stage, and tissue [10,42,43,45]. Wide geographic separation and pronounced environmental divergence induce physiological and biochemical shifts that modulate metabolite accumulation [43,46]. For example, *Z. nitidum* from Guangdong showed a markedly higher alkaloid content than that from Guangxi [8], while leaves from Qujing, Yunnan, contained significantly more hesperidin and total flavonoids than material from Guangdong or Guangxi [6]. Harvest timing further alters composition [2,43,45]. Monoterpenoids in the leaf EO increased to a maximum at ZN-2 (39.75%) and subsequently declined, a pattern paralleled by the accumulation of chlorogenic acid and luteoloside in *L. japonica* [44] and by total alkaloids in *Z. nitidum* [2]. Consequently, ZN-3 represents the optimal stage for maximal sesquiterpenoid content, whereas ZN-2 provides the highest monoterpenoid level.

### 3.5. FTIR Fingerprints, HCA, and PCA of Z. nitidum

**Figure 5:**
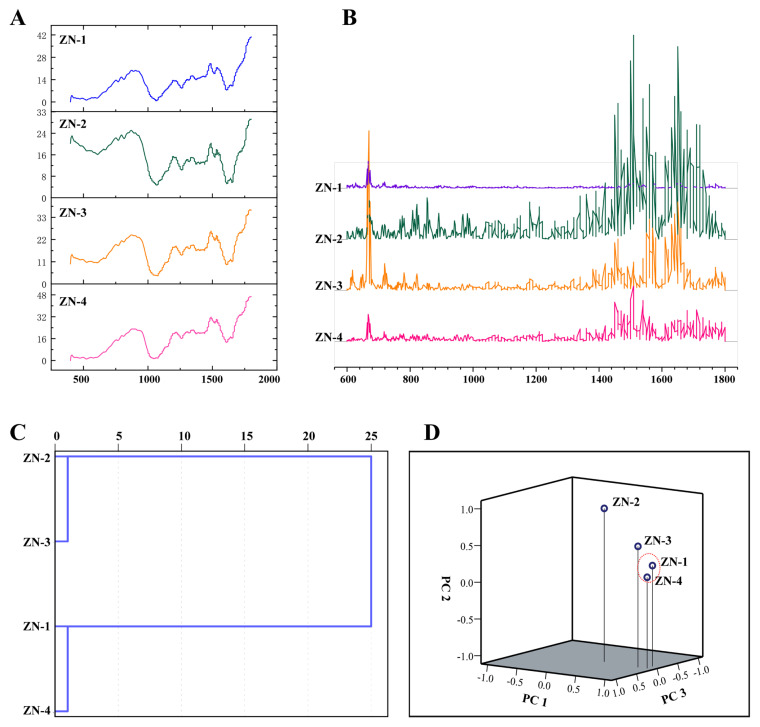
*Zanthoxylum nitidum* leaves at different developmental stages: (**A**) IR spectra, (**B**) second-derivative IR spectra, (**C**) cluster dendrogram, and (**D**) PCA score scatter plot.

FTIR spectra represent the superposition of functional groups present in the sample [8,32]. Analysis of the FTIR fingerprints of *Z. nitidum* leaf powders from four developmental stages revealed the 1800~600 cm^−1^ region to be the most characteristic (Figure 5A). Within this window, the spectra of all four groups highly overlapped, reflecting both chemical complexity and similarity that precluded visual differentiation. Second-derivative spectra (Figure 5B), which enhance resolution, amplify weak bands, and suppress baseline drift [6,8], showed a common peak near 660 cm^−1^ attributable to out-of-plane bending of aromatic C-H [47]. Marked divergence was observed at 1420~1800 cm^−1^, corresponding to C=O stretching, C=C stretching, aliphatic C-H bending, and aromatic C=C skeletal vibrations [47]; of these, ZN-2 exhibited the largest amplitude, followed by ZN-3 and ZN-4.

To further characterize the spectroscopic signatures of *Z. nitidum* leaf powders across developmental stages, chemometric tools including HCA and PCA were applied to the absorbance data. The dendrogram (Figure 5C) resolved the four samples into two primary clusters: the first was formed by ZN-2 and ZN-3, linked by a small distance coefficient (<5); the second comprised ZN-1 and ZN-4, also with a comparably low distance coefficient.

Although hierarchical cluster analysis accurately classified the objects at various distance levels, it failed to reveal relationships between non-adjacent samples [48,49]. To clarify these interrelationships, the second-derivative FTIR spectra spanning 4000~400 cm^−1^ were selected as the analytical window and subjected to PCA. Figure 5D presents the three-dimensional scatter plot derived from PC1, PC2, and PC3. PC1 explained 61.58% of the total variance and was therefore the dominant component, whereas PC2 and PC3 accounted for 20.78% and 12.75%, respectively. The cumulative contribution of the first three components reached 95.11%, indicating that only 4.89% of the spectral information was lost; consequently, these components captured the vast majority of the original data. Each sample occupied a distinct position in the principal component space, enabling effective discrimination among the four developmental stages. Consistent with the cluster analysis, ZN-1 and ZN-4 clustered together within the red dashed circle in the 3D plot, whereas ZN-2 and ZN-3 were located farther away, with ZN-3 slightly closer to the red circle, mirroring the differences observed in the second-derivative spectra (Figure 5B). Thus, the 3D scatter plot not only provided accurate classification but also offered an intuitive visualization of the relationships among samples. Both cluster analysis and PCA have been widely employed to investigate and differentiate medicinal materials such as *Z. nitidum*, *C. cassia*, and *Stellaria dichotoma* var. *lanceolata* Bunge [2,4,8,10,50,51].

## 4. Conclusions

This study is the first to integrate cytological, analytical–chemical, and chemometric approaches to systematically elucidate the ontogeny of secretory cavities and the temporal accumulation of essential oils in *Z. nitidum* leaves at four developmental stages. (1) Secretory cavities in the leaflets were unequivocally identified as the marginal-tooth-slit type. (2) Each cavity exclusively comprised a spherical domain and originated strictly through a schizogenous mechanism. (3) The EO yield displayed a unimodal trajectory, peaking at stages ZN-2 and ZN-3. GC-MS profiling resolved 65 principal constituents, among which sesquiterpenoids reached their maximum content at ZN-3, and monoterpenoids were most abundant at ZN-2. (4) The second-derivative FTIR spectra of leaf powders exhibited pronounced inter-stage differences; HCA and PCA enabled reliable classification and identification of the developmental stages based on their bioactive signatures, underscoring the robustness and practical utility of the methodology. Collectively, these findings establish a comprehensive cytological and analytical–chemical framework for quality control and rational utilization of *Z. nitidum* leaves, thereby providing a scientific basis for the full exploitation of this medicinal resource.

## Figures and Tables

**Table 1:** Chemical composition of *Zanthoxylum nitidum* leaves at different developmental stages.

No.	Compound Name	RI	Relative Content (%)				Identification
			ZN-1	ZN-2	ZN-3	ZN-4	
1	2-Hexenal	850	—	2.95 ± 0.059	—	1.25 ± 0.072	GC-MS, RI
2	3-Hexen-1-ol	859	—	0.49 ± 0.047	—	0.91 ± 0.054	GC-MS, RI
3	*trans*-2-Hexenol	861	—	1.56 ± 0.064	—	0.72 ± 0.030	GC-MS, RI
4	β-Thujene	936	0.26 ± 0.010	—	—	—	GC-MS, RI
5	α-Pinene	948	2.18 ± 2.18	—	—	—	GC-MS, RI
6	(+)-Sabinene	983	4.70 ± 2.35	—	1.37 ± 0.040	—	GC-MS, RI
7	α-Myrcene	991	1.14 ± 0.57	0.70 ± 0.019	—	—	GC-MS, RI
8	Isocarvestrene	1018	—	2.27 ± 0.023	—	—	GC-MS, RI
9	Eucalyptol	1031	—	1.76 ± 0.017	—	0.34 ± 0.019	GC-MS, RI
10	α-Tolualdehyde	1044	—	0.36 ± 0.027	—	—	GC-MS, RI
11	(*E*)-β-Ocimene	1048	1.78 ± 0.049	1.31 ± 0.0090	—	0.96 ± 0.037	GC-MS, RI
12	Terpinolen	1082	0.31 ± 0.0030	—	—	—	GC-MS, RI
13	Linalool	1101	3.38 ± 0.078	8.17 ± 0.043	0.39 ± 0.010	2.24 ± 0.089	GC-MS, RI
14	Terpinen-4-ol	1162	—	0.33 ± 0.055	—	—	GC-MS, RI
15	(-)-α-Terpineol	1197	0.32 ± 0.00	2.74 ± 0.035	—	0.62 ± 0.017	GC-MS, RI
16	Decanal	1208	0.21 ± 0.00	—	—	—	GC-MS, RI
17	2-Isopropyl-5-methyl-3-cyclohexen-1-one	1251	—	—	—	3.76 ± 1.88	GC-MS, RI
18	Piperitone	1262	2.11 ± 0.020	22.47 ± 0.057	0.68 ± 0.68	2.00 ± 2.00	GC-MS, RI
19	2-Undecanone	1294	18.00 ± 0.092	0.56 ± 0.012	0.57 ± 0.0090	—	GC-MS, RI
20	δ-EIemene	1347	—	0.21 ± 0.11	0.70 ± 0.70	0.91 ± 0.46	GC-MS, RI
21	(-)-α-Cubebene	1361	0.45 ± 0.00	—	0.25 ± 0.0030	0.23 ± 0.0030	GC-MS, RI
22	α-Copaene	1375	0.31 ± 0.31	—	—	—	GC-MS, RI
23	α-Ylangene	1377	0.61 ± 0.31	—	0.47 ± 0.0030	—	GC-MS, RI
24	Elemene	1402	—	0.20 ± 0.20	3.03 ± 0.077	—	GC-MS, RI
25	β-Caryophyllene	1430	15.37 ± 0.013	6.51 ± 0.046	8.78 ± 0.11	9.80 ± 0.12	GC-MS, RI
26	β-Copaene	1437	9.93 ± 0.095	—	15.92 ± 0.057	15.87 ± 0.035	GC-MS, RI
27	γ-Elemene	1444	0.90 ± 0.040	0.45 ± 0.028	2.27 ± 0.069	1.41 ± 0.12	GC-MS, RI
28	Isogermacrene D	1448	—	—	0.45 ± 0.00	—	GC-MS, RI
29	(+)-Aromadendrene	1456	—	—	0.38 ± 0.00	—	GC-MS, RI
30	α-Humulene	1471	6.01 ± 0.023	2.52 ± 0.0090	5.61 ± 0.012	4.00 ± 0.030	GC-MS, RI
31	γ-Muurolene	1489	0.99 ± 0.015	—	0.96 ± 0.0030	—	GC-MS, RI
32	Germacrene D	1497	—	8.35 ± 0.093	—	—	GC-MS, RI
33	2-Tridecanone	1456	0.43 ± 0.015	—	—	—	GC-MS, RI
34	Bicyclogermacrene	1495	4.52 ± 0.022	2.65 ± 0.056	9.61 ± 0.042	5.87 ± 0.055	GC-MS, RI
35	α-Farnesene	1505	1.83 ± 0.059	0.51 ± 0.25	4.01 ± 0.14	2.29 ± 0.069	GC-MS, RI
36	α-Amorphene	1512	0.33 ± 0.0070	—	—	—	GC-MS, RI
37	γ-Amorphene	1518	0.37 ± 0.0070	—	0.89 ± 0.012	0.20 ± 0.017	GC-MS, RI
38	δ-Amorphene	1521	2.43 ± 0.024	1.07 ± 0.032	3.87 ± 0.038	1.78 ± 0.013	GC-MS, RI
39	Germacrene B	1561	1.17 ± 0.064	0.72 ± 0.047	2.80 ± 0.12	1.99 ± 0.10	GC-MS, RI
40	*trans*-Nerolidol	1564	4.84 ± 0.084	8.55 ± 0.082	17.39 ± 0.035	15.55 ± 0.039	GC-MS, RI
41	(-)-Spathulenol	1572	0.25 ± 0.006	1.06 ± 0.035	1.31 ± 0.015	1.13 ± 0.022	GC-MS, RI
42	Caryophyllene oxide	1578	—	—	0.40 ± 0.40	1.26 ± 0.029	GC-MS, RI
43	(-)-Globulol	1580	0.94 ± 0.023	0.88 ± 0.050	0.78 ± 0.39	—	GC-MS, RI
44	(-)-Epicedrol	1585	0.70 ± 0.024	0.65 ± 0.019	—	1.14 ± 0.012	GC-MS, RI
45	Cedrol	1589	—	—	1.40 ± 0.021	—	GC-MS, RI
46	Humulene epoxide II	1606	—	—	0.22 ± 0.0070	—	GC-MS, RI
47	Junenol	1618	0.35 ± 0.012	—	0.24 ± 0.00	—	GC-MS, RI
48	Isospathulenol	1630	—	—	0.29 ± 0.0060	0.31 ± 0.0090	GC-MS, RI
49	T-Muurolol	1640	0.89 ± 0.012	0.75 ± 0.019	1.24 ± 0.020	0.92 ± 0.028	GC-MS, RI
50	α-Cadinol	1653	1.59 ± 0.037	2.39 ± 0.078	2.05 ± 0.047	2.02 ± 0.027	GC-MS, RI
51	β-Sinensal	1693	—	—	—	0.64 ± 0.035	GC-MS, RI
52	Mintsulfide	1742	—	—	0.32 ± 0.010	—	GC-MS, RI
53	α-Sinensal	1765	—	0.46 ± 0.023	—	0.49 ± 0.015	GC-MS, RI
54	m-Camphorene	1944	—	0.23 ± 0.023	—	—	GC-MS, RI
55	n-Hexadecanoic acid	1959	—	3.06 ± 0.041	—	4.81 ± 0.26	GC-MS, RI
56	Conjugated linoleic acid	2060	—	0.56 ± 0.56	—	—	GC-MS, RI
57	Linoelaidic acid	2065	—	1.04 ± 0.52	—	—	GC-MS, RI
58	Phytol	2111	—	—	0.31 ± 0.020	1.37 ± 0.050	GC-MS, RI
59	Linolenic acid	2159	—	1.18 ± 0.052	—	1.73 ± 0.024	GC-MS, RI
60	11,14-Eicosadienoic acid	2250	—	—	—	1.84 ± 0.065	GC-MS, RI
Total			89.60	89.67	88.96	90.36	

Data is expressed as mean ± SEM. RI, retention indices; GC-MS, gas chromatography–mass spectrometry; —, not detected.

## Data Availability

All data from this study have been published in the manuscript. There is no additional data available.
